# Comparative Study of Protein Aggregation Propensity and Mutation Tolerance Between Naked Mole-Rat and Mouse

**DOI:** 10.1093/gbe/evac057

**Published:** 2022-04-28

**Authors:** Savandara Besse, Raphaël Poujol, Julie G. Hussin

**Affiliations:** Département de Biochimie et Médecine Moléculaire, Faculté de Médecine, Université de Montréal, Québec, Canada; Centre Robert-Cedergren en Bioinformatique et Génomique, Université de Montréal, Québec, Canada; Institut de Cardiologie de Montréal, Québec, Canada; Institut de Cardiologie de Montréal, Québec, Canada; Département de Médecine, Faculté de Médecine, Université de Montréal, Québec, Canada

**Keywords:** naked mole-rat, longevity, aging, protein homeostasis, protein aggregation propensity, mutation tolerance

## Abstract

The molecular mechanisms of aging and life expectancy have been studied in model organisms with short lifespans. However, long-lived species may provide insights into successful strategies for healthy aging, potentially opening the door for novel therapeutic interventions in age-related diseases. Notably, naked mole-rats, the longest-lived rodent, present attenuated aging phenotypes compared with mice. Their resistance toward oxidative stress has been proposed as one hallmark of their healthy aging, suggesting their ability to maintain cell homeostasis, specifically their protein homeostasis. To identify the general principles behind their protein homeostasis robustness, we compared the aggregation propensity and mutation tolerance of naked mole-rat and mouse orthologous proteins. Our analysis showed no proteome-wide differential effects in aggregation propensity and mutation tolerance between these species, but several subsets of proteins with a significant difference in aggregation propensity. We found an enrichment of proteins with higher aggregation propensity in naked mole-rat, and these are functionally involved in the inflammasome complex and nucleic acid binding. On the other hand, proteins with lower aggregation propensity in naked mole-rat have a significantly higher mutation tolerance compared with the rest of the proteins. Among them, we identified proteins known to be associated with neurodegenerative and age-related diseases. These findings highlight the intriguing hypothesis about the capacity of the naked mole-rat proteome to delay aging through its proteomic intrinsic architecture.

**Significance**

The molecular mechanisms behind naked mole-rat longevity are still poorly understood. Here, we address how the proteome architecture can help delay the onset of aging in naked mole-rat by studying properties that modulate protein aggregation. We identify ∼1,000 proteins with significant differences in aggregation propensity and mutation tolerance involved in processes known to be dysfunctional during aging. These findings highlight how evolutionary adaptations in protein aggregation in distinct biological processes could explain naked mole-rat longevity.

## Introduction

Understanding the mechanism of aging and life longevity is a major biological problem. The hallmarks of aging describe the dysfunction of several biological processes such as genomic instability, telomere attrition, loss of protein homeostasis (proteostasis), epigenetic alterations, mitochondrial dysfunction, cellular senescence, stem cell exhaustion, deregulated nutrient-sensing pathways, and altered intercellular communication (López-Otín et al. 2013). The aggravation of these hallmarks usually leads to an early manifestation of aging while their amelioration contributes to its delay and an increase in healthy lifespan. However, all the hallmarks are not yet fully supported by experimental interventions that succeeded in improving aging and extending lifespan. The genetics behind the hallmarks of aging have been identified through genetic perturbation studies in multiple model organisms such as yeast, nematodes, flies, and mice (reviewed in Singh et al. 2019; Taormina et al. 2019). These model organisms have been critical in our understanding of aging thanks to their short lifespan that aids tractable experimentation, relatively cheap maintenance, and possibilities for genetic manipulation. However, there is a need to study organisms with longer lifespans to understand the mechanisms behind their longevity better. Recent whole-genome sequencing efforts allowed the study of organisms with a longer lifespan. Cross-species “omics” studies of these long-lived species, such as transcriptomic, metabolic, and lipidomic profiles associated with long-lived species, highlighted molecular signatures that could be important to aging (reviewed in Ma and Gladyshev 2017; Tian et al. 2017). One notable example is the naked mole-rat, the longest-lived rodent among those with a known maximum lifespan and a model organism for studies on healthy aging and longevity (Buffenstein and Ruby 2021). Indeed, this organism presents attenuated age-related changes, suggesting the presence of antiaging mechanisms contributing to its longevity (Buffenstein 2005). Several comparative studies between naked mole-rat and mice reported significant differences in their maintenance of protein homeostasis. Naked mole-rats show high oxidative damage levels from young ages (Andziak et al. 2006). Still, their ubiquitinylated proteins are maintained at lower levels at both young and old ages, suggesting less accumulation of damaged and misfolded proteins during aging (Pérez et al. 2009). The low levels of damaged and misfolded proteins could also be explained by their high proteasome activity (Rodriguez et al. 2012). Taken together, these observations emphasize the importance to study the general principles contributing to the robustness of protein homeostasis in the naked mole-rat. Nevertheless, these general principles have not been established at the proteome level. Thus, in this paper, we propose identifying the proteomic features that contribute to protein homeostasis maintenance.

In naked mole-rats, several works have studied the molecular key players of protein homeostasis and their potential role in rodent longevity. For example, proteostasis-centered theories of aging propose that aging results from the decline of quality control systems involved in protein synthesis, degradation, and chaperoning that normally contribute to protein turnover (Balch et al. 2008; Powers et al. 2009; Proctor and Lorimer 2011; Taylor and Dillin 2011). Proteostasis is essential for protein stability through the protection of their structures and functions against environmental perturbations. Impaired proteostasis leads to the appearance of phenotypic aging markers and age-related diseases such as Alzheimer’s and Parkinson’s diseases, known to be characterized by the accumulation of protein aggregates of specific proteins (Irvine et al. 2008; Powers et al. 2009; Hipp et al. 2019). Indeed, there is an increase in the expression of chaperones with higher proteasome and autophagy activities in naked mole-rat (Tian et al. 2017). From a system biology perspective, the maintenance of proteostasis is essential for delaying the onset or slowing down the process of aging (Koga et al. 2011). In addition, the protein aggregates are processed by quality control systems such as chaperones and protein degradation pathways (proteasome and autophagy) (Morimoto and Cuervo 2009). These mechanisms are robust in young individuals but tend to decline with age, leading to an increase of protein aggregates within the cell, thus participating in the dysfunction of multiple biological processes (Labbadia and Morimoto 2015). A recent study in *Caenorhabditis elegans* describes the proteostasis decline with age and observed an exponential increase of protein aggregates in old cells (Santra et al. 2019).

Our study focuses on intrinsic protein properties that could contribute to proteostasis maintenance by reducing the formation of protein aggregates. Causes of protein aggregation can arise from protein features and cell features. Protein aggregation propensity is a protein sequence feature that characterizes the ability of the protein to aggregate and is estimated based on the physicochemical properties of the amino acid (AA) sequence. However, this property of intrinsic aggregation propensity alone does not fully determine whether a protein will aggregate in vivo, which is determined by confounding cellular factors (e.g., cellular concentration, recruitment by chaperones). Whether a sequence that has high aggregation propensity will in fact aggregate will need to account for cellular features. In the cell, cumulative damage through nonenzymatic posttranslational modifications from reactions with metabolites or reactive oxygen species (Golubev et al. 2017), leads to protein instability, and subsequently to the formation of protein aggregates. Alternatively, the formation of protein aggregates could result from destabilizing mutations. The accumulation of somatic mutation burden has been proposed as a driver of aging (Vijg 2014). Several studies previously demonstrated the importance of mutation accumulation in the onset of aging and the reduction of lifespan (Lodato et al. 2018; Lee et al. 2019). However, it is still unclear whether the accumulation of mutation would contribute to the formation of protein aggregates. To tackle this question, we also propose to study “mutation tolerance” or the ability of proteins to tolerate the potential effects of mutations on their aggregation propensity.

Here, we performed a comparative analysis on protein aggregation propensity and the mutation tolerance between the naked mole-rat and the mouse. From the study of these two properties, we aim to understand how they might contribute to explaining the difference in lifespan between these two species. First, we estimated their aggregation propensity between the two species at the level of whole-protein sequences (entire open reading frames [ORFs]) and individual folding domains. We performed a random and exhaustive computational mutagenesis to estimate the mutation tolerance of these proteins. We found that although there is no global difference in aggregation propensity in the proteome shared between naked mole-rat and the mouse, we identified groups of proteins that significantly differ in their aggregation propensity. This observation holds both at the level of individual domains and the level of entire protein sequences. By performing gene set enrichment analyses, we retrieve several biological processes; some of them were already reported to be potentially involved in the naked mole-rat longevity, notably processes associated with the immune system. We also highlight their inflammation’s versability, as we found proteins with high and low aggregation propensities from this process. We also identified proteins, previously reported as involved in neurodegenerative diseases in human, that have not yet been considered as aging gene markers. Furthermore, these subsets of proteins have different distributions of mutation tolerance in the naked mole-rat, but not in the mouse, suggesting specific adaptations of these properties in the longest-lived rodent.

## Results

### Analysis of the Orthologous Proteome Shared Between Naked Mole-Rat and Mouse

**Fig. 1.**
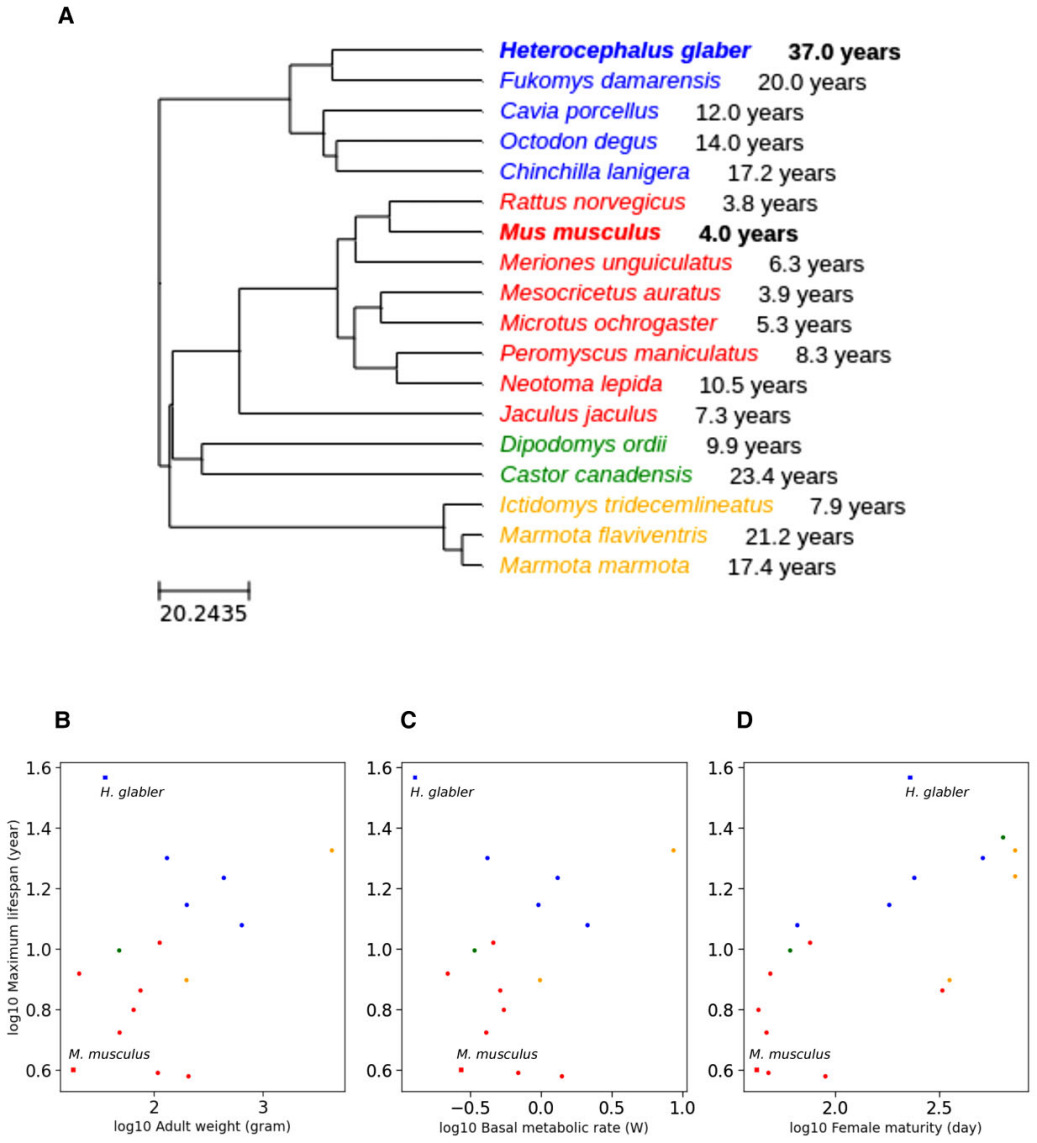
Maximum lifespan variation across rodents. (*A*) Phylogenetic distribution of rodent species with known maximum lifespan. The tree was generated with TimeTree using rodent species with known maximum lifespan. Four groups were identified according to their closest common ancestor. Mouse (*Mus musculus*) and naked mole-rat (*Heterocephalus glaber*), highlighted in bold, are the selected organisms for our comparative study, as they have a drastic difference of maximum lifespan. Mouse can live up to 4 years, whereas naked mole-rat can live up to 37 years. Rodent maximum lifespan compared with (*B*) adult weight, (*C*) basal metabolic rate, and (*D*) female maturity, for the rodents mentioned in (*A*). The maximum lifespan, adult weight, female maturity, and metabolic rate data are extracted from the *AnAge* database. All values were log10-transformed. Mouse and naked mole-rat are represented with a square shape.

To check the lifespan variability across rodents (fig. 1*A*), we collected the maximum lifespan data available in the *AnAge* database (Tacutu et al. 2018) and retrieved information for 18 species. Furthermore, we extracted several metrics describing life-history traits such as body mass, basal metabolic rate, and female maturity available in *AnAge,* previously shown to be correlated with maximum lifespan in mammals (Fushan et al. 2015). On the reconstructed rodent phylogenetic tree, we observed that indeed the naked mole-rat is the longest-lived rodent (Ruby et al. 2018) and shares a common ancestor with other rodents living more than 12 years (fig. 1*A*, first group from top). This group is separated from a larger monophyletic group, which include a large cluster (fig. 1*A, second group from top*) with rodents with a shorter maximum lifespan, <10 years, including mouse. The remaining two groups (fig. 1*A*, third and fourth group from top) contain a low number of species with no clear tendency in their maximum lifespan. We plotted life-history traits metrics against the maximum lifespan (fig. 1*B*–*D*), confirming that the naked mole-rat is an outlier from the rest of the rodents. These observations support the fact that the naked mole-rat is an appropriate organism to study aging because of its unexpectedly long lifespan among rodents, in contrast to the mouse that is a good representative for short-lived species.

To identify the general principles behind the naked mole-rat longevity, we compared the orthologous proteome shared between naked mole-rat and mouse. The mouse has a well-curated and annotated genome and has also been extensively studied in the field of aging (Mitchell et al. 2015). Our comparative analysis between naked mole-rat and mouse focuses on 13,806 ortholog pairs collected from the orthologous mapping database *Inparanoid* (see Materials and Methods). We considered two properties among these orthologous proteins, specifically: (1) their aggregation propensity and (2) their mutation tolerance, to determine if they could partly explain the higher maintenance of protein homeostasis in naked mole-rat compared with the mouse. To study these properties within the two species, we estimated the aggregation propensity of the ortholog pairs using the software *Tango* (Fernandez-Escamilla et al. 2004) (see Materials and Methods), which scores the per-residue aggregation propensity of protein sequences. With this tool, the property of protein aggregation propensity is accurately predicted on proteins with no transmembrane regions; therefore, we excluded the transmembrane proteins (see Materials and Methods), leaving a total of 9,522 ortholog protein pairs.

Since different regions of an ORF could have different folding properties, the aggregation propensity scores were also computed at the domain level. To do so, we retrieved 19,413 annotated domains available for 8,475 proteins (see Materials and Methods). Moreover, we looked more closely at a specific subset of proteins, the chaperone client proteins, which are the proteins interacting with chaperones in known protein–protein interaction networks. This subset is composed of 1,298 protein pairs (see Materials and Methods).

### Specific Subsets of Proteins Display Significant Differences in Aggregation Propensity

**Fig. 2.**
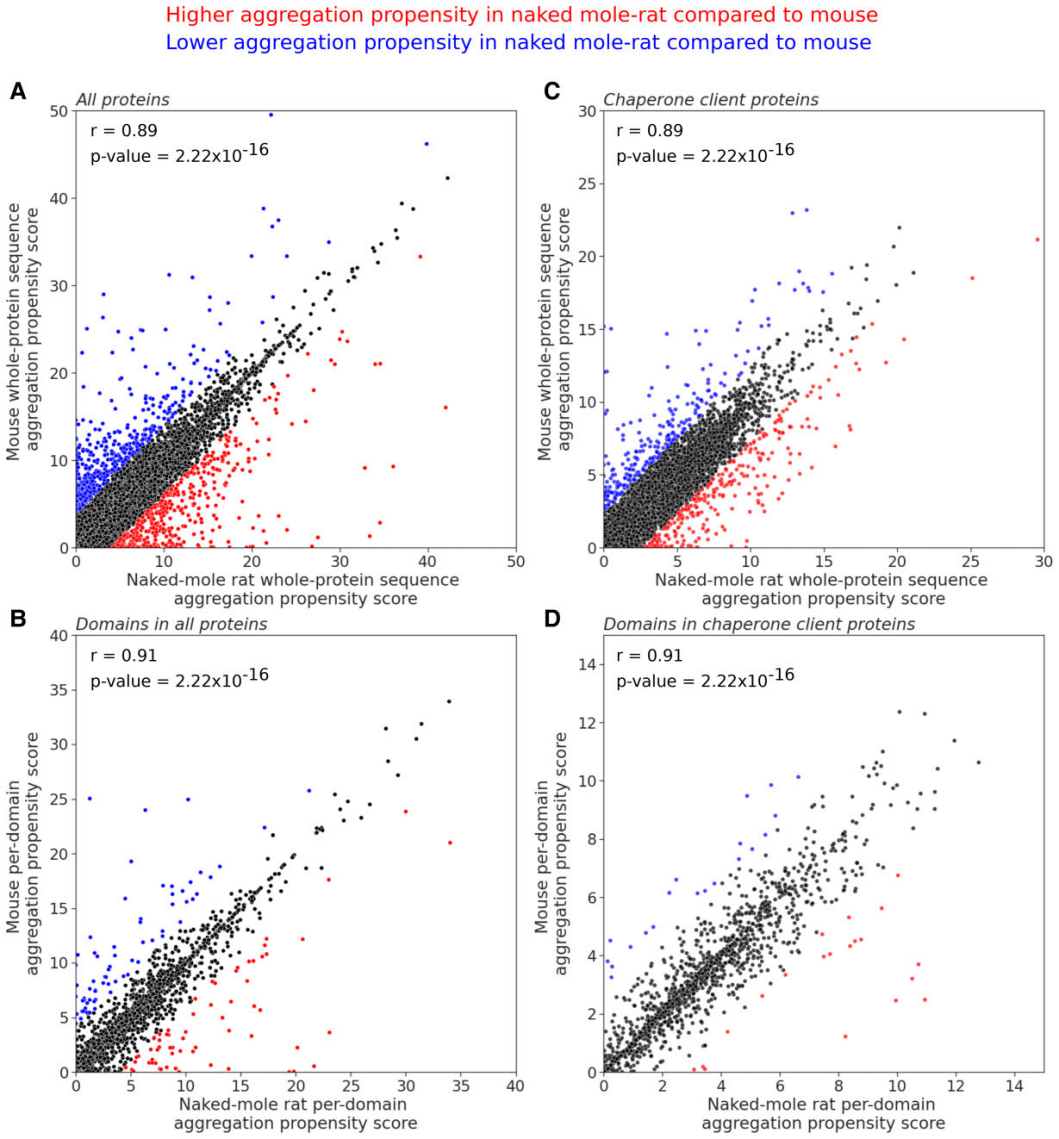
Study of aggregation propensity in naked mole-rat and mouse. Comparison of aggregation propensity scores in orthologous proteins from naked mole-rat and mouse. Each point represents an ortholog pair. Whole-protein sequence aggregation propensity scores (Agg_P_) for the (*A*) whole data set (*n* = 9,522) and (*B*) subset of chaperone client proteins (*n* = 1,298). Per-domain aggregation propensity scores (Agg _D_) for the (*C*) whole data set (*n* = 19,413 domains) and (*D*) subset of chaperone client proteins (*n* = 3,126 domains). See Materials and Methods for details on calculations Agg_D_ and Agg_P_. Pearson’s correlation coefficients (*r*) between the naked mole-rat and mouse aggregation propensity scores are reported.

The accumulation of protein aggregates is potentially toxic to cells (Stefani and Dobson 2003) and results from the decline of protein homeostasis. Protein aggregation tends to increase with age and initiate amyloid-beta aggregation in *nematodes* and mice (Groh et al. 2017). Since such protein aggregates are found in specific tissues and cause age-related diseases such as Alzheimer’s and Parkinson’s diseases, we asked whether the systematic presence of proteins with a higher chance to aggregate within the cells could be correlated to the onset of aging. In the naked mole-rat, despite high levels of oxidation, they maintain low rates of ubiquitylated proteins (Pérez et al. 2009), suggesting a reduced formation of protein aggregates. To identify if there is a proteome-wide difference in protein aggregation propensity between naked mole-rat and mouse, we first estimated the protein aggregation propensity on the ortholog proteins using Tango (see Materials and Methods). For a given protein sequence, this approach estimates the per-residue aggregation propensity scores based on their physicochemical properties with specific environmental parameters. With these scores, we computed two metrics: (1) an aggregation score for the whole-protein sequence and (2) an aggregation score for each annotated domain of the proteins (see Materials and Methods). We compared the aggregation scores between naked mole-rat and mouse, in the whole-protein sequence, and their domains (fig. 2).

Overall, whole-protein sequence propensity scores are low (Naked mole-rat Agg_P_ = 3.48 ± 2.77, Mouse Agg_P_ = 3.37 ± 2.73) and per-domain aggregation propensity scores have higher variance than whole-protein sequence (Naked mole-rat Agg _D_ = 3.79 ± 4.60, Mouse Agg _D_ = 3.76 ± 4.57). We observed a high correlation in aggregation propensity between naked mole-rat and mouse at the whole-protein sequence (*r* = 0.89, *P*-value = 2 × 10^−16^) and the domain (*r* = 0.91, *P*-value= 2 × 10^−16^), indicating no proteome-wide global differences in aggregation propensity between these two species (fig. 2*A* and *B*). In parallel, we focused on the chaperone client proteins to see if they have specific aggregation propensity and mutation tolerance compared with the rest of the proteins since they interact with the chaperones. Their whole-protein sequence and per-domain aggregation propensity scores are also low (Naked mole-rat Agg_P_ = 3.48 ± 2.37, Mouse Agg_P_ = 3.37 ± 2.31). We observed a high correlation in aggregation propensity, as in the all-proteins data set, at the whole-protein sequence level (*r* = 0.89, *P*-value = 2 × 10^−16^) and the domain level (*r* = 0.91, *P*-value = 2 × 10^−16^) (fig. 2*C* and *D*), suggesting that chaperone client proteins do not differ in terms of aggregation propensity between these two species.

We computed differences in aggregation propensity (ΔAgg) to identify proteins differing significantly between the species. Altogether, we found 269 proteins (including 20 chaperone clients) with higher whole-protein sequence aggregation propensity (*z*-scores > 2, see Materials and Methods) in naked mole-rat compared with mouse, and 247 proteins (including 21 chaperone clients) with lower aggregation propensity (*z*-scores < −2). In proteins with annotated domains (*n* = 8,475), we found 904 protein domains with significantly different aggregation propensity scores within 754 different proteins. Specifically, 452 protein domains (including 63 domains from chaperone clients) have higher aggregation propensity (*z*-scores > 2) in the naked mole-rat compared with mouse, and 452 protein domains (including 70 domains from chaperone clients) have lower aggregation propensity (*z*-scores < −2). In total, in combining the whole-protein sequence and per-domain analyses, we identified 1,155 distinct proteins with differences in their aggregation propensity. Additionally, we see no significant difference when comparing the distribution of ΔAgg *z*-scores from chaperone client proteins to proteome-wide values for the whole-protein sequence (*P*-value = 0.72, Student’s *t*-test) and per-domain analyses (*P*-value = 0.90, Student’s *t*-test). The proportion of proteins with a significant difference of aggregation propensity is similar in chaperone client proteins and the other proteins, indicating the chaperone client subset is not enriched in proteins with a significant difference of aggregation propensity between the naked mole-rat and mouse.

### Function of Proteins with a Significant Difference of Aggregation Propensity

**Fig. 3.**
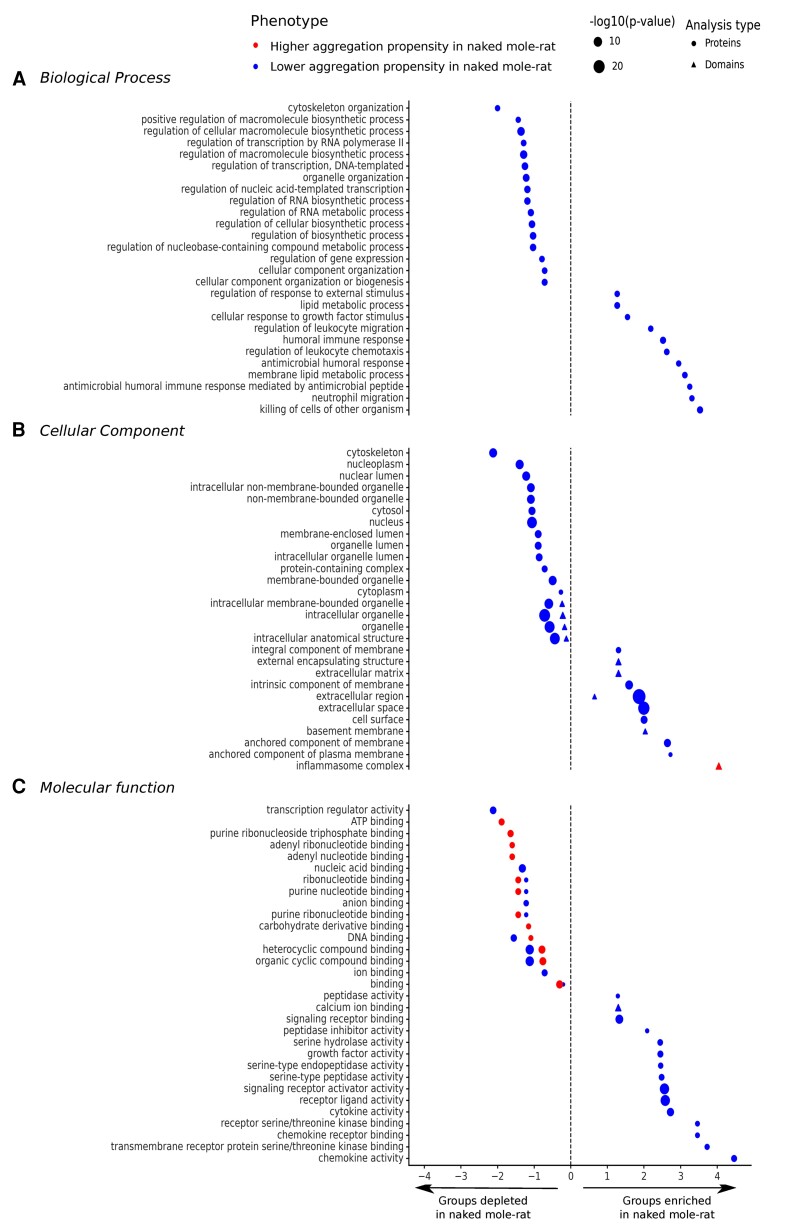
Significant GO terms associated with domains and proteins with higher and lower aggregation propensity in naked mole-rat. Log2 fold enrichment (FE) values indicate which GO terms are depleted (log2 FE < 0) or enriched (log2 FE > 0) in proteins (circle shape) and domains (triangle shape) with a higher or a lower aggregation propensity in naked mole-rat. The GO terms are grouped by categories: (*A*) Molecular Function, (*B*) Cellular Component, and (*C*) Biological Process. The size of the dots is proportional to their −log10 *P*-values. Only GO terms with at least five proteins and FDR < 0.05 are shown.

We investigated the over- and under-representation of specific Gene Ontology (GO) annotation terms associated with protein subsets with either significantly high or low aggregation propensity in naked mole-rat (see Materials and Methods). We computed and sorted enrichment scores associated with each GO term (fig. 3). We found enriched or depleted groups having proteins with low aggregation propensity in naked mole-rat (in blue). These groups are associated with GO terms within Biological Process (fig. 3*A*) and Cellular Component (fig. 3*B*) categories. Depleted groups in *Biological Process* category are cell organization (5 × 10^−7^ < *P*-value < 7 × 10^−5^), regulation of different macromolecule biosynthesis (2 × 10^−8^ < *P*-value < 8 × 10^−5^) and regulation of gene expression (*P*-value = 3 × 10^−5^). Proteins with significantly low aggregation propensity are under-represented in these processes. In contrast, enriched groups are related to immune response (*P*-value = 8 × 10^−6^) and lipid metabolism (*P*-value = 4 × 10^−5^). The amid ceramidase (ASAH1), an enzyme involved in lipid metabolism, is associated with age-related diseases (Parveen et al. 2019). Depleted groups in *Cellular Component* category are intracellular compartments, while enriched groups are membrane (*P*-value = 5 × 10^−9^ and *P*-value = 7 × 10^−5^) and extracellular components (fig. 3*B*), such as the extracellular matrix (*P*-value = 1 × 10^−28^) and the cell surface (*P*-value = 4 × 10^−7^). Notably, in these compartments, we found numerous metalloproteases from the matrixin family such as MMP3, MMP10, MMP13, and MMP19 containing several haemopexin repeats; MMP7 and MMP25 with a peptidase M10 domain. These metalloproteases can degrade proteins from the extracellular matrix.

Additionally, we noticed that proteins in the inflammasome complex (*P*-value = 3 × 10^−6^) contain domains with significantly high aggregation propensity in the naked mole-rat. Particularly, we identified the peptidase C14 domain of CASP-1 (Caspase 1) and CASP-12 (Caspase 12) from the caspase family, the NOD2-WH domain of NLRP-1A (Nod-Like Receptor Pyrin domain-containing 1A), NLRP-3 (Nod-Like Receptor Pyrin domain-containing 3), and NLRP-6 (Nod-Like Receptor Pyrin domain-containing 6), the functional domain of GSDMDC1 (Gasdermin Domain-Containing protein 1), and the CARD domain of NLRC4 (Nod-Like Receptor CARD domain-containing protein 4). All these proteins are involved in inflammation. Surprisingly, only two of the seven identified inflammasome complex proteins have higher aggregation propensity in naked mole-rat compared with mouse (*z*-scores > 2) at the whole-sequence level, despite all of them having domains with higher aggregation propensity in naked mole-rat compared with mouse (supplementary fig. S2, Supplementary Material online) (Besse_et_al_SM.docx). When investigating further the domains with higher aggregation propensity of the inflammasome proteins, we observed that similar protein domain families are shared across the  ORFs. The domain peptidase C14 is restricted to the caspase family (supplementary fig. S2*C*, Supplementary Material online) (Besse_et_al_SM.docx), and usually has higher aggregation propensity scores in naked mole-rat than mouse. However, we observed that only peptidase C14-containing proteins with a significant difference of aggregation propensity between species are involved in the formation of inflammasome complexes, such as CASP-1, CASP-12, and CASP-4. The CARD domain is also shared among proteins but the difference of domain aggregation propensity is less consistent across proteins with the CARD domain (supplementary fig. S2*D*, Supplementary Material online) (Besse_et_al_SM.docx).

The lack of enriched GO terms for the subset of proteins with high aggregation propensity in naked mole-rat than in mouse across all GO categories suggests this may be a random group of proteins. However, in the *Molecular Function* category (fig. 3*C*), we identified depleted groups in proteins with significantly high aggregation propensity related to ATP binding and its subcategories (*P*-value = 1 × 10^−5^). The associated proteins with these functions have a more conserved aggregation propensity than expected by chance. Interestingly, we found depleted groups containing both proteins with higher and lower aggregation propensity in naked mole-rat, related to various binding functions. This observation supports the fact that proteins with specific and well-defined molecular functions are generally more structurally conserved across species and are less likely to have significant differences of aggregation propensity between species. Nevertheless, only one group (calcium ion binding, *P*-value = 3 × 10^−6^) contains proteins with differences of aggregation propensity from domains. The other enriched groups from the *Molecular Function* category have proteins with different enzymatic activities (serine-type peptidase, *P*-value = 3 × 10^−5^; serine hydrolase, *P*-value = 3 × 10^−5^). Among them, we identified Chymotrypsin-C, which contributes to proteolysis, the breakdown of proteins as polypeptides. Finally, we found enriched groups of proteins associated with chemokine and cytokine activity (*P*-value = 1 × 10^5^; *P*-value = 1 × 10^−7^, respectively). We identified several members of the chemokine family, the immunoglobulin receptor IL-40, the interferon-alpha IFNA13, the Cerberus, and Wnt-2b proteins from the Wnt pathway. All annotations of the proteins associated with specific GO terms are shown in supplementary table S5, Supplementary Material online (Besse_et_al_SM.xlsx).

Furthermore, the distribution of the number of proteins per GO terms within each category (supplementary fig. S1, Supplementary Material online) (Besse_et_al_SM.docx) is similar for chaperone client proteins and the other proteins, except for the ones associated with immune response and extracellular components (marked with an asterisk, corrected *P*-values < 0.05, *χ*^2^ test, supplementary fig. S1, Supplementary Material online) (Besse_et_al_SM.docx), indicating there are few or no proteins with lower aggregation in naked mole-rat that need chaperones to fold in these groups.

### Proteins with Lower Aggregation Propensity in Naked Mole-Rat Better Tolerate Mutations

Finally, we explored the somatic mutation theory of aging by studying mutation tolerance in naked mole-rat and mouse orthologous proteins. This theory hypothesizes that mutation accumulation is an essential player in the onset of aging (Kennedy et al. 2012) and influences longevity. We designed a large-scale in silico mutagenesis experiment by generating all possible 1-nucleotide mutations on gene sequences for 9,346 protein pairs (of length below 10,000 AAs) and then estimated the aggregation propensity of these mutants (see Materials and Methods). The difference between the aggregation propensity from mutated sequences and the aggregation propensity from the original sequences allows us to predict if a substitution would increase, maintain, or decrease this property. Assuming that proteins would preferably tolerate substitutions that do not significantly change their aggregation propensity, we derive a mutation tolerance score defined as a ratio of the number of substitutions with no change on the aggregation propensity (Mutational Agg _P_ = 0) divided by the total number of generated substitutions (strict mutation tolerance, see Materials and Methods, eq. 6). These values range from 0 to 1, representing weak to strong tolerance to substitutions. In this definition, being tolerant correspond to the ability of the protein to strictly maintain the property of aggregation propensity.

**Fig. 4.**
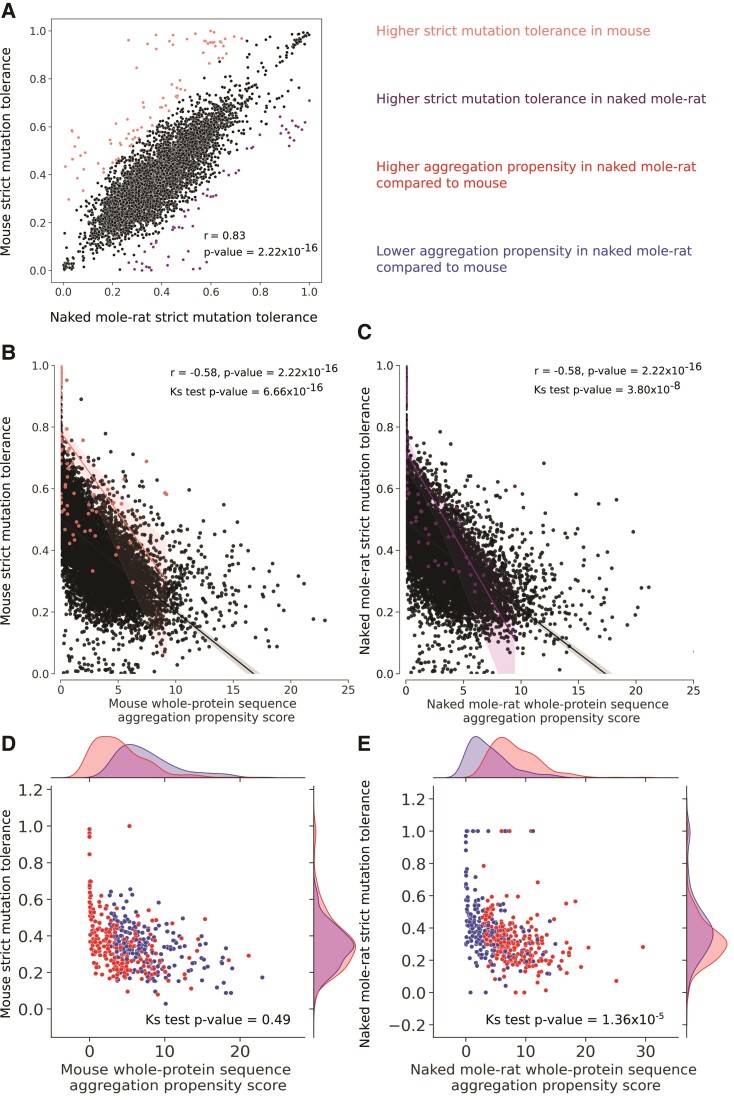
Study of mutation tolerance in naked mole-rat and mouse. (*A*) Comparison of mutation tolerance scores in orthologous proteins between naked mole-rat and mouse (*n* = 7,939 proteins). Correlation between mutation tolerance and whole-protein sequence aggregation propensity scores in (*B*) mouse and (*C*) naked mole-rat. Protein pairs with significant differences in mutation tolerance are colored, using the color code from (*A*). Pearson’s correlation (*r*) between mutation tolerance and aggregation propensity is reported in both organisms. The Kolmogorov–Smirnov (KS) test is used to assess the difference of distribution between proteins with mutation tolerance scores similar in mouse and naked mole-rat, and the ones which are different. Scatterplots of mutation tolerance against whole-protein sequence aggregation propensity scores in (*D*) mouse and in (*E*) naked mole-rat, restricted to the subsets of proteins identified with significant difference of aggregation propensity (*n* = 510 proteins). The KS test is used to assess differences in mutation tolerance distributions between the two subsets in each organism.

This score allows us to study the relationship between whole-protein sequence aggregation propensity and strict mutation tolerance of orthologous proteins in the two rodents (fig. 4). There is a high correlation between strict mutation tolerance scores between the naked mole-rat and the mouse (*r* = 0.83, *P*-value = 2 × 10^−16^), suggesting no global difference in strict mutation tolerance between their proteomes (fig. 4*A*). In both species, we observed a negative correlation between the sequence aggregation propensity and the strict mutation tolerance (*r* = −0.58, *P*-value = 2 × 10^−16^), suggesting that proteins with a low aggregation propensity tend to be more resistant to substitutions. Moreover, it also suggests that proteins with high aggregation propensity scores contain more residues with nonzero aggregation propensity scores, suggesting that these residues might be more affected by random mutations (fig. 4*B* and *C*). Importantly, we identified subsets of proteins with significant differences in strict mutation tolerance between the species (fig. 4*A*). We tested whether proteins with significant differences in strict mutation tolerance between the species have a similar aggregation propensity to the rest of the data set. The distribution of aggregation propensity for proteins with higher strict mutation tolerance compared with the distribution of other proteins is significantly different in both species (fig. 4*B*, Mouse *P*-value = 1 × 10^−19^; fig. 4*C* Naked mole-rat *P*-value = 2 × 10^−15^, Kolmogorov–Smirnov test), with proteins with higher strict mutation tolerance in a species having lower aggregation propensity compared with the rest of the proteins. This result implies that the proteins with low aggregation propensities better tolerate mutations which is not surprising, given that our strict mutation tolerance score itself is based on the whole-protein sequence aggregation propensity. We investigated the function of these proteins by performing an enrichment analysis as previously described, but no specific GO term was under- or overrepresented in these subsets.

Moreover, we investigated the strict mutation tolerance scores of the proteins with higher and lower aggregation propensity in naked mole-rat compared with a mouse. In the mouse (fig. 4*D*), the distributions of strict mutation tolerance scores between higher and lower aggregation propensity proteins are not significantly different (*P*-value = 0.49, Kolmogorov–Smirnov test), indicating that the distributions of the strict mutation tolerance of the two subsets are similar. However, in naked mole-rat (fig. 4*E*), we find a significant difference in the strict mutation tolerance scores between higher and lower aggregation subsets (*P*-value = 2 × 10^−8^, Kolmogorov–Smirnov test). In naked mole-rat, proteins with lower aggregation propensity better tolerate substitutions than proteins with higher aggregation propensity. These proteins are found in biological processes or pathways shown in figure 3, which we will discuss as potential players toward naked mole-rat longevity.

We next tested if our results hold up when mutations that decrease aggregation propensity are included in the computation of another mutation tolerance score (referred to as lenient mutation tolerance, see Materials and Methods, eq. 8). In this definition, we include not only neutral mutations (no change in aggregation propensity), but also mutations that would decrease aggregation propensity (Mutational Agg _P_ ≤ 0). Indeed, it could be assumed that these mutations would be beneficial and should therefore be considered as tolerated. With this alternative definition, although the lenient mutation tolerance of naked mole-rat and mouse are less correlated (*r* = 0.77 vs. *r* = 0.83), the main results described above hold (supplementary fig. S3, Supplementary Material online) (Besse_et_al_SM.docx). In mouse, the distributions of “lenient” mutation tolerance scores between higher and lower aggregation propensity proteins are still not significantly different (*P*-value = 0.56, Kolmogorov–Smirnov test), whereas in the naked mole-rat (supplementary fig. S3*C*, Supplementary Material online) (Besse_et_al_SM.docx), a similar difference in the lenient mutation tolerance scores between higher and lower aggregation subsets is seen (*P*-value = 0.06, Kolmogorov–Smirnov test). Since including beneficial mutations weakens the difference seen in the naked mole-rat, we further explored the specific signature associated with beneficial mutations (supplementary fig. S3*D*, Supplementary Material online) (Besse_et_al_SM.docx). We computed the proportion of beneficial mutations in proteins in both species (see Materials and Methods) and observed a low correlation between proportions from naked mole-rat and mouse (*r* = 0.43, *P*-value = 2.22 × 10^−16^), suggesting that the proteins that can possibly improve their property of aggregation propensity with beneficial mutations are not the same in naked mole-rat and mouse. Furthermore, in both species (supplementary fig. S3*E* and *F*, Supplementary Material online) (Besse_et_al_SM.docx), we find a significant difference in the distribution of these proportions between higher and lower aggregation subsets (*P*-value = 1.19 × 10^−12^ for mouse, *P*-value = 1.07 × 10^−14^, Kolmogorov–Smirnov test), with proteins with high aggregation propensity in a given species having a higher potential for accumulating beneficial mutations compared with proteins with low aggregation propensity in the respective species. Similar trends have been previously observed for protein stability (Serohijos et al. 2012).

### Evolutionary Changes Specific to Naked Mole-Rat Influence Local Differences in Aggregation Propensity in ATX Proteins

**Fig. 5.**
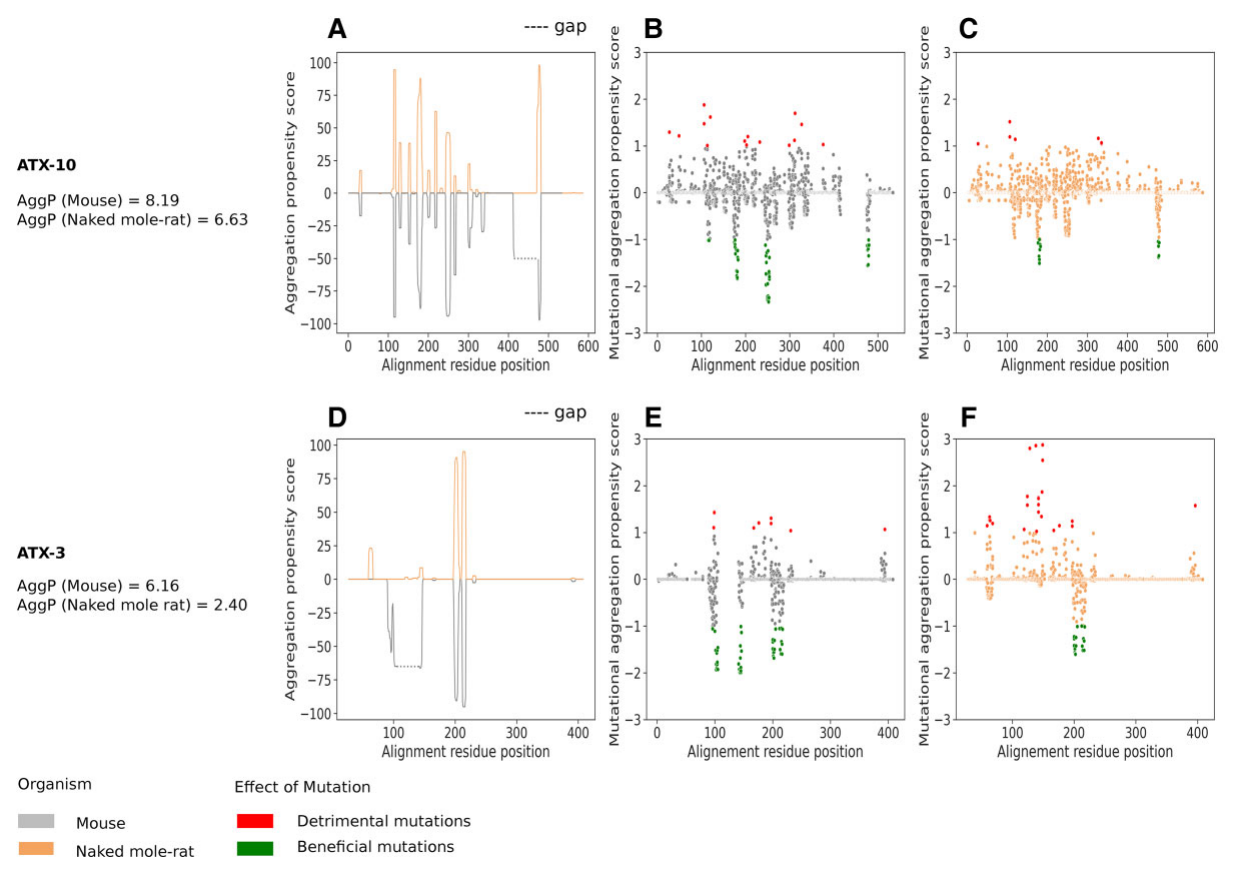
Aggregation propensity and mutational aggregation propensity profiles of ATX proteins. Aggregation propensity profile along the positions from the sequence alignment of the two rodent protein sequences for (*A*) ATX-10 and (*D*) ATX-3 proteins. We plot the opposite of the mouse aggregation value to facilitate comparison. Regions with gap are represented with a dashed line. Mutational aggregation profile of mouse and naked mole-rat for (*B* and *C*) ATX-10 and (*E* and *F*) ATX-3 proteins. Beneficial and detrimental mutations are annotated (top for detrimental, Mutational Agg _P_ > 1; bottom for beneficial, Mutational Agg _P_ < −1).

Among the proteins we identified with significant difference of aggregation propensity between the species, several of them have been reported to be associated with human age-related diseases. Specifically, Ataxin-10 (ATX10) and Ataxin-3 (ATX3) are both responsible for different forms of spinocerebellar ataxia in humans, a type of neurodegenerative disease. Both proteins have a lower whole-protein sequence aggregation propensity in naked mole-rat, compared with mouse (ATX-10: 6.63 [naked mole-rat] < 8.19 [mouse]; ATX-3: 2.40 [naked mole-rat] < 6.16 [mouse]). We investigated the origin of these differences of aggregation propensity in these ATX proteins by comparing the distribution of their per-residue aggregation propensity scores and mutational aggregation propensity score between the two species along the aligned sequences (fig. 5).

In ATX-10 (fig. 5*A*–*C*) and in ATX-3 (fig. 5*D*–*F*), regions with high peaks of aggregation propensity (Agg > 50) are co-localized with negative mutational aggregation hotspots in both species, whereas residues with positive mutational aggregation regions are found in low aggregation propensity regions (Agg ∼ 0), with fewer random mutations resulting in increased aggregation (detrimental mutations) than resulting in decreased aggregation (beneficial mutations). This is in line with the idea that regions of high aggregation propensity would benefit more from mutations than low aggregation propensity regions would be affected by mutations, suggesting that the low aggregation propensity regions are possibly in an optimal state favoring protein robustness.

In both proteins, we observed insertions in naked mole-rat (dashed line, fig. 5*A* and *D*), which are not found in any other rodent species (blast *e*-value <0.001, see Materials and Methods). Interestingly, in both cases, these insertions are located within a mouse aggregation propensity peak (positions 416–474 of the ATX10 alignment and positions 106–140 of the ATX3 alignment). Overall, naked mole-rat and mouse ATX-10 (fig. 5*A*) have similar aggregation propensity profiles, meaning that the residues with high and low aggregation propensity are located in the same regions, but with mouse having higher values between position 200 and 300. In this region, we indeed observe a hotspot of random mutations beneficial in mouse only (fig. 5*B*), indicating that this region has the potential to acquire lower aggregation propensity in mouse, whereas in naked mole-rat (fig. 5*C*), the region’s aggregation propensity is not significantly improved by mutations. In the case of ATX-3 (fig. 5*D*), naked mole-rat and mouse have distinct aggregation propensity profiles before AA 150, with naked mole-rat having lower aggregation propensity compared with mouse in the region of the naked mole-rat-specific insertion. In mouse only, we observed hotspots of beneficial mutations on both sides of the insertion (fig. 5*E*), and in naked mole-rat, random mutations within the insertion tend to significantly increase aggregation propensity (fig. 5*F*). This result indicates that the insertion is likely the determining factor of the decrease in aggregation propensity in ATX3 in naked mole-rat compared with mouse. This region is already stabilized for mutations. Interestingly, this insertion is located in the functional domain Josephin that is known to contribute to ubiquitin chain binding and cleavage for ATX-3 (Nicastro et al. 2009). In the case of the ATX10 insertion, which split a PF09759 domain seen in the mouse sequence into two subdomains in the naked mole-rat sequence, we did not observe detrimental mutations in the insertion in the naked mole-rat, and both species show an increase in aggregation propensity at the insertion breakpoint.

## Discussion

Aggregation propensity and mutation tolerance are two intrinsic properties of proteins that could contribute to the better maintenance of protein homeostasis. In this study, we designed a computational strategy to estimate these properties at the scale of the whole-proteome in naked mole-rat and mouse using a comparative genomic framework. Among their orthologous proteome (*n* = 9,522 proteins), we did not identify global differences in aggregation propensity, but about 1,000 proteins showed significant differences from their domains or their whole-protein sequences. Our analyses specifically study chaperone client proteins to determine whether this subset has differing intrinsic properties but did not find significant differences. Previous studies have shown that chaperone client proteins evolve slower and have a lower aggregation propensity compared with nonclient proteins (Victor et al. 2020). Still, our study shows that these properties remain similar between naked mole-rat and mouse. As for caveats, we inferred the naked mole-rat and mouse chaperone clients from human orthologs based on information reported in the BioGRID database. The data from this database do not necessarily indicate actual chaperone dependence. Therefore, it is possible that the subset of proteins we defined as chaperone client proteins is highly incomplete or does not interact with chaperones in naked mole-rat and/or in mice. Moreover, we do not specify which specific chaperones were interacting with those chaperone client proteins, which may also bias the results.

From the gene-enrichment analysis, we observed that the proteins of naked mole-rat with less aggregation propensity are overrepresented mainly in the extracellular compartments, within several specific biological processes related to immune response and lipid metabolism, and have functions associated with binding and protein degradation. The proteins with more aggregation propensity are not enriched in a particular biological process, except in the inflammasome complex, known to contain aggresomal complexes. Among the proteins we identified with significant differences in aggregation propensity, we identified several previously known proteins in neurodegenerative and age-related diseases. For instance, ATX3 is a poly-glutamine tract-containing protein that contributes to cytoskeleton organization and is involved in protein inclusion bodies (Burnett and Pittman 2005). The accumulation of ATX3 in brain cells causes a proteostasis impairment that leads to the Machado–Joseph disease, or spinocerebellar ataxia-3 (Dantuma and Herzog 2020). Particularly, ATX3 is associated with double-stranded DNA binding. Previously, the study of ATX3-mutant in mouse brain cells showed an impairment of DNA repair efficiency, leading to the accumulation of DNA damage (Gao et al. 2015). ATAX10 was also identified here, which is associated with pentanucleotide disorder SCA10, (Bampi et al. 2017). Identifying lower aggregation propensity in these poly-glutamine proteins in naked mole-rat could contribute to resistance toward certain types of neurodegenerative diseases, leading to premature death (Dantuma and Herzog 2020). Moreover, we also identified proteins related to lipid metabolism with lower aggregation propensity in naked mole-rat, such as the acid ceramidase ASAH1. This protein is involved in the intra-lysosomal ceramide homeostasis and is known to be associated with Alzheimer’s disease, cancer, and diabetes (Parveen et al. 2019). Furthermore, a recent study highlighted specific lipidic signatures in naked mole-rat that confer neuroprotective mechanisms against oxidative damage (Frankel et al. 2020). The lower aggregation propensity of the lipid metabolism proteins may contribute to protein stability and discharge of quality control systems of proteostasis.

Our study also highlighted the versatility of the aggregation propensity within inflammation pathways in naked mole-rats. Indeed, these rodents have a unique immune system able to better resist bacterial infection. They have a unique myeloid cell subset that highly expressed genes for the antimicrobial response (Hilton et al. 2019). Genes involved in the NOD-like receptor signaling pathway can activate pyroptosis, which is cell death after exposure to a bacterial infection. Interestingly, the NLRP-3 inflammasome pathway, which we found to have a higher aggregation propensity at the level of protein domains in our study, is known to be regulated by the ubiquitin system. However, the exact molecular mechanisms of its noncanonical activation remain unclear (Lopez‐Castejon 2019). The increase of domain aggregation propensity within proteins associated with the inflammasome complex might explain their affinity with the ubiquitin system; however, we note that this result could be explained by specific domains (e.g., peptidase C14) overrepresented in proteins involved in the formation of inflammasome complexes. Moreover, naked mole-rat’s immune system is more frequently solicited during bacterial infection than in the mouse (Cheng et al. 2017). Our study observed that proteins with chemokine and cytokine activity have significantly lower aggregation propensity. This suggests that the intrinsic properties of these naked mole-rat proteins adapt to be less prone to aggregate.

We also identified several metalloproteases having domains with lower aggregation propensity in the naked mole-rat. Metalloproteases are known to degrade extracellular matrix proteins. Interestingly, the naked mole-rats highly produce the high-molecular-mass hyaluronan (Tian et al. 2013), a component of the extracellular matrix, known to have anti-inflammatory properties (Takasugi et al. 2020). These proteins might facilitate the hyaluronan turnover and balance the proinflammatory responses from the high activity of the inflammasome. Recently, two studies highlighted the importance of MMP13 as a therapeutic target for Alzheimer’s and Parkinson’s disease (Zhu et al. 2019; Sánchez and Maguire-Zeiss 2020). Tight regulation of inflammatory responses in naked mole-rat seems essential to maintain protein homeostasis, particularly during bacterial infection. Naked mole-rats are known to maintain proteasomal proteolytic activities in their late stages of life (Pérez et al. 2009). These adaptations could indirectly promote healthy aging in naked mole-rat, increasing its maximum lifespan.

Mutation tolerance is another intrinsic property of proteins that could contribute to maintaining protein homeostasis. It indicates the ability of the protein to maintain its aggregation propensity despite mutations. We used the difference of aggregation propensity between mutated and wild-type sequences to estimate whether a substitution event in the coding sequence would later drastically change or not the aggregation propensity of a protein. In the definition of our mutation tolerance score, synonymous substitutions favor protein stability and avoid the formation of protein aggregates. Despite no global differences in mutation tolerance between the two species’ proteomes, proteins with lower aggregation propensity in naked mole-rat better tolerate mutation than proteins with higher aggregation propensity. Such a difference is not seen in the mouse, which suggests these proteins in naked mole-rat have intrinsic properties that slow down the overload of the quality control systems of proteostasis, thus might contribute to its longevity.

We further studied ATX-10 and ATX-3, from ATX-family, known to be associated with the neurodegenerative disease, spinocerebellar ataxia in humans. We were able to highlight evolutionary events that occurred only in naked mole-rat sequences and that helped to improve the aggregation propensity in that species. We observed peaks of aggregation propensity close to insertion breakpoints in the two ATX proteins. Notably, for ATX-3, we observed that an insertion in a functional domain decreases the aggregation propensity profile of the naked mole-rat compared with the mouse sequence, suggesting that this event contributes to the stability of the protein. The combined observation of the aggregation propensity and mutational aggregation profiles of these specific regions thus informs on the consequences of evolutionary events such as insertions on aggregation propensity and protein stability. These regions are candidates that could be further studied for optimized protein design toward stability. It would be also interesting to see if other proteins with significant differences in aggregation propensity might also contain insertion events specific to naked mole-rat. Another future study could try to identify if the specific insertions in naked mole-rat are systematically co-localized with aggregation propensity, as well as in other species. These analyses are of course not exhaustive but are good examples of the potential use of our different metrics to perform comparative analyses of proteins that could explain the difference in protein stability between the two species, with possible implications for differences in lifespan.

Studying the diversity of lifespan within eukaryotes with comparative genomic approaches requires well-curated genome assemblies and reliable maximum lifespan measurements. In this study, we restricted our analysis to two species from the same taxonomic order, with a drastic difference of maximum lifespans, to identify the proteomic features explaining their lifespan difference. Working with closed-related species helps to identify subsets of proteins associated explicitly with biological processes related to longevity in the two species, without taking account of the complications arising from comparing from evolutionary-distant species. Although these results could be specific to rodents, the pathways and genes identified in this study are known to be shared across eukaryotes. Therefore, our study is a step toward a more extensive investigation of these properties across species. In addition to restricting the comparative analysis to only two species, our study has several limitations. First, we only focused on orthologous proteins shared between naked mole-rat and mouse, ignoring proteins unique to naked mole-rat, which could also contribute to its extended longevity. Second, to predict the aggregation propensity of the proteins shared between naked mole-rat and mouse, we used the *Tango* software, which is a predictive approach that heavily relies on the physicochemical properties of the AA sequences and their likelihood to be involved in the formation of beta-sheets structures participating in functional folding. This approach performs well to predict the aggregation propensity of globular proteins (Linding et al. 2004), which resulted in the exclusion of transmembrane and membrane proteins from our analyses. Moreover, the aggregation propensity scores are predicted for a given set of environmental parameters. They may not represent the dynamic range of aggregation propensity scores that the proteins could adopt in different tissues. Alternative bioinformatics methods to estimate aggregation propensity based on AA sequences are implemented as web server tools (Santos et al. 2020), incompatible with our high-throughput computational strategy for estimating mutation tolerance by generating billions of sequences that could only be processed promptly using a command-line software. Therefore, *Tango* allowed us to build a systematic and highly efficient pipeline to estimate the aggregation propensity of ∼10,000 proteins in two different organisms. This large-scale experiment is unfeasible to achieve in vitro. However, further molecular investigations will be necessary to validate the role of the identified less aggregation-prone proteins in naked mole-rat in the context of aging. Finally, to validate whether the patterns we identified regarding aggregation propensity and mutation tolerance are not only specific to the comparison of naked mole-rat to mouse, these patterns will need to be more systematically confirmed by comparing long-lived versus short-lived rodents. The challenge of this strategy will be to properly define the long-lived and short-lived groups and verify if the phylogenetic relationships in each group are equally distributed. The use of longevity quotient (Austad and Fischer 1991), which indicates whether a species has an average lifespan or is unusually long- or short-lived relative to its body size, could be used to distinct the groups with extreme longevity.

In conclusion, we investigated the peculiarity of naked mole-rat longevity by studying specific intrinsic properties of the proteome that influence the maintenance of proteostasis. Our study highlighted a trade-off in the regulation of inflammation responses in the naked mole-rat, directly encoded in the AA composition of the proteins as it relates to its propensity to aggregation. We also identified several proteins with lower aggregation propensity compared with the mouse that has been found to characterize neurodegenerative or age-related diseases in humans. Our findings propose the existence of a successful strategy encoded in the naked mole-rat proteome architecture to delay aging through better maintenance of protein homeostasis in the longest-lived rodent.

## Materials and Methods

### Definition of the Orthologous Data Set and Subsets

Orthologous sequences are homologous sequences that share similarities from a speciation event. The orthologous AA sequences shared between naked mole-rat and mice were retrieved using the *Inparanoid* algorithm (version 4.1) (Remm et al. 2001) with default parameters. As initial inputs, we use the naked mole-rat and mouse latest proteome assemblies, downloaded from Uniprot (https://www.uniprot.org/, accessed April 2019). The *Inparanoid* algorithm performs a reciprocal best-hit search to cluster the orthologous and in-paralog proteins, to identify the orthologous groups between the two species. For our analysis, each orthologous group was represented by a pair of proteins with the highest mutual best hit score, yielding 13,806 orthologous pairs. Mouse and naked mole-rat Uniprot protein identifiers are available in supplementary table S2, Supplementary Material online (Besse_et_al_SM.xlsx). To assess the quality of these orthologous pairs, we computed their local alignments with Matcher (Waterman and Eggert 1987; Huang and Miller 1991) and collected the percentage of similarity and the percentage of gaps within the pairwise alignments. Orthologous pairs with a percentage of similarity below 60% or a percentage of gaps above 20% were removed, altogether keeping a total of 13,513 pairs.

For the estimation of aggregation propensity from Tango software (see below), we excluded transmembrane proteins. To identify the proteins with transmembrane regions to exclude, we first parsed mouse gene annotations available in the proteome FASTA file and defined the ones containing the keyword “transmembrane” as transmembrane proteins and excluded them. Additionally, we also predicted transmembrane regions in the remaining sequences with TMHMM (Krogh et al. 2001). All mouse and naked mole-rat proteins with at least one transmembrane region predicted were removed, restricting our analyses to 9,522 protein pairs. We also collected their associated protein-coding nucleotide sequences for our computational large-scale mutagenesis analysis (see below). Moreover, we identified a specific subset, containing all the proteins known to interact with chaperone proteins. For this specific data set, we used the human chaperone client proteins (annotated with their ENSEMBL identifiers) from a recent study (Victor et al. 2020) to infer the mouse chaperone clients. The human ENSEMBL identifiers were converted to their corresponding Uniprot identifiers for mapping them toward the mouse Uniprot ortholog identifiers. Similarly, we then mapped the mouse Uniprot identifiers to the naked mole-rat ortholog identifiers. This specific subset of orthologs is composed of 1,298 protein pairs.

### Identification of Protein Domains in Naked Mole-Rat and Mouse

To obtain mouse and naked mole-rat domain definitions, we first collected mouse domain information from the Pfam database (http://pfam.xfam.org/, version 33.1). Within a given protein, we considered any peptide as a functional domain when their entire sequence matched domain annotations, corresponding to the start and end positions in PFAM protein alignments. For the naked mole-rat, the domain definitions were inferred using the reciprocal best hit method where the mouse annotated domains are used as reference. We collected a total number of 19,413 annotated domains available for 8,475 protein pairs, representing 89% of our initial data set.

### Phylogenetic Tree and Data Related to Longevity

The evolutionary distances between rodent species were determined using *TimeTree* (Kumar et al. 2017), through the available webserver. This method retrieved all existing phylogenetic trees for the given species and provided the concatenation of these trees to determine the median time when species diverged. These phylogenetic trees were built based on gene alignments. The available information on maximum lifespan, adult weight, female maturity, and metabolic rate for rodent species was retrieved from the *AnAge* database (Tacutu et al. 2018, build 14) and are given in supplementary table S1, Supplementary Material online (Besse_et_al_SM.xlsx). We reported more recent maximum lifespans for naked mole-rat (Buffenstein and Ruby 2021) and damaraland naked mole-rat (Rodriguez et al. 2016).

### Computation of Aggregation Propensity Scores

To predict the propensity of proteins to aggregate, we used the Tango software (Fernandez-Escamilla et al. 2004). Tango assigns per-residue aggregation propensity scores based on the AA physicochemical properties. For each orthologous protein pair, we computed the per-residue aggregation score with Tango for each sequence independently and then calculated their whole-protein sequence aggregation and domain aggregation. Per-domain aggregation score is defined as the sum of the per-residue aggregation propensity score for a defined functional domain divided by the domain length (Agg*_D_*, eq. 1). The whole-protein sequence aggregation propensity score is defined as the sum of per-residue aggregation propensity scores for the entire sequence divided by the protein length (Agg*_P_*, Equation 2).

(1) $${\mathrm{Agg}}_{D}=\frac{\begin{array}{c} \sum \;\mathrm{Per}\text{-}\mathrm{residue}\;\mathrm{Aggregation}\;\mathrm{propensity}\;\\ \mathrm{score}\;(\mathrm{for}\;\mathrm{domain}\;\mathrm{sequence}) \end{array}}{\mathrm{Domain}\;\mathrm{length}}$$

 

(2) $${\mathrm{Agg}}_{P}=\frac{\begin{array}{c} \sum \;\mathrm{Per}\text{-}\mathrm{residue}\;\mathrm{Aggregation}\;\mathrm{propensity}\\ \mathrm{score}\;(\mathrm{for}\;\mathrm{whole}\text{-}\mathrm{protein}\;\mathrm{sequence}) \end{array}}{\mathrm{Protein}\;\mathrm{length}}$$

### Identification of Proteins with Significant Difference of Aggregation Propensity

To compare mouse and naked mole-rat protein aggregation propensity scores, we computed their difference at the domain (ΔAgg*_D_*, eq. 3) and the whole-protein sequence (ΔAgg*_P_*, eq. 4) levels with the following formulas:

(3) $${\mathrm{\Delta Agg}}_{D}=\;{\mathrm{Agg}}_{D\;;\mathrm{Naked}\text{-}\mathrm{Mole}\;\mathrm{rat}}\;\;\text{–}\;{\mathrm{Agg}}_{D\;;\mathrm{Mouse}}$$

 

(4) $${\mathrm{\Delta Agg}}_{P}=\;{\mathrm{Agg}}_{P\;;\mathrm{Naked}\text{-}\mathrm{Mole}\;\mathrm{rat}}\;\;\text{–}\;{\mathrm{Agg}}_{P\;;\mathrm{Mouse}}$$

The difference of aggregation propensity scores was normalized to obtain *z*-scores. Proteins with *z*-scores exceeding 2 times the standard deviation are considered significantly different. Both for whole-sequence and domain aggregation propensity analyses, two groups were defined as: (1) proteins with ΔAgg *z*-scores > 2 being considered to have a higher aggregation in naked mole-rat compared with mouse and (2) proteins with ΔAgg *z*-scores < −2 being considered to have a lower aggregation in naked mole-rat compared with mouse.

### Functional Enrichment Analyses

With the previously identified subsets of proteins, we investigated the cellular components, molecular functions, and biological processes from GO annotations, these proteins are over- or under-represented. To do so, we used hypergeometric tests implemented on the Panther database (Mi et al. 2019). As the protein annotations for naked mole-rat were not proposed in the database, we used the annotations from the mouse, assuming the naked mole-rat proteins have similar annotations to their mouse orthologs. The subsets from the domain analysis were compared with the set of proteins with annotated domains within the shared proteome (*n* = 8,475). The subsets from the whole-protein sequence analyses were compared with all the proteins of the shared proteome (*n* = 9,522). Raw *P*-values of Fisher’s exact tests were computed to identify the gene ontologies significantly over- or under-represented for each subset, corrected by a false discovery rate (FDR). Only GO terms associated with at least five proteins are shown in figure 3. The entire list of GO terms with FDR < 0.05 and, for the domain and the whole-protein sequence analyses, are available in supplementary tables S3 and S4, Supplementary Material online, respectively (Besse_et_al_SM.xlsx). The list of proteins within the groups and their annotations are available in supplementary table S5, Supplementary Material online (Besse_et_al_SM.xlsx).

To identify which GO terms where the chaperone client proteins are differently distributed compared with the rest of the proteins, we computed *χ*^2^ tests, corrected by a Benjamini/Hochberg FDR.

### Quantification of Protein Mutation Tolerance

This quantification of mutation tolerance was initially performed on 9,522 protein pairs. However, 176 proteins (mostly proteins with more than 10,000 AAs) were removed as the calculation of their mutation tolerance score was too computationally expensive, thus, reducing the data set to 9,346 protein pairs. We also removed protein pairs where naked mole-rat coding sequences were truncated, obtaining a final data set of 7,939 proteins.

We designed a large-scale in silico mutagenesis experiment to estimate the mutation tolerance of the proteins shared between naked mole-rat and mouse. Specifically, the mutation tolerance score is a ratio from 0 to 1 that quantifies the ability of a protein to tolerate mutations. We mutated one nucleotide at a time within the DNA sequence to all three other possible nucleotide mutations (self-substitution is excluded). For example, for a coding sequence of *X* nucleotides, we would generate *X*^3^ possible substitutions that would engender *X*^3^ mutated sequences. All these DNA sequences are then translated into AA sequences. We kept only nonredundant protein sequences (resulting from nonsynonymous changes), different from the wild-type sequence (WT), for predicting their protein aggregation propensity using Tango, as described in the section *Computation of aggregation scores*. Whole-protein sequence aggregation scores for mutated (MT) sequence were then computed and are used to calculate the difference of aggregation propensity (mutational aggregation propensity score, Mutational Agg _P_—eq. 5) between MT and WT sequences:

(5) $$\mathrm{Mutational}\;\mathrm{Agg}{\;}_{P}=\;{\mathrm{Aggregation}}_{P\;;\mathrm{MT}}\;\;\text{–}\;{\mathrm{Aggregation}}_{P\;;\mathrm{WT}}$$

We defined three categories of proteins, according to their change in aggregation propensity:

1. Mutational Agg_P_ = 0: No change in aggregation propensity of the mutated sequence
2. Mutational Agg_P_ > 1: High increase in aggregation propensity of the mutated sequence
3. Mutational Agg_P_ < −1: High decrease in aggregation propensity of the mutated sequence

For a given protein, these scores were used to define their mutation tolerance. It calculated the ratio of the number of mutations with no impact on protein aggregation normalized by the number of all possible mutations (Strict Mutation tolerance, eq. 6). The total number of mutations corresponds to the number of protein sequences which result from a nonsynonymous substitution that does alter the length of the protein. Therefore, we exclude the truncated sequences resulting from the change of the first methionine of the AA sequence and the ones that contain premature codon stop by checking that the lengths of the wild-type protein sequence and the mutated sequence are equal.

(6) $$\begin{array}{rl} & \mathrm{Strict}\;\mathrm{mutation}\;\mathrm{tolerance}\; \end{array}=\frac{\mathrm{Number}\;\mathrm{of}\;(\mathrm{Mutational}\;\mathrm{Agg}\;P\;=\;0)}{\mathrm{Total}\;\mathrm{number}\;\mathrm{of}\;\mathrm{mutations}}$$

For identifying proteins with a significant difference in their strict mutation tolerance, we calculated the difference of strict mutation tolerance between naked mole-rat and mouse (ΔMutTol).

(7) $$\begin{array}{r} \Delta \mathrm{MutTol}={\mathrm{Strict}\;\mathrm{Mutation}\;\mathrm{tolerance}}_{\mathrm{Naked}\text{-}\mathrm{mole}\;\mathrm{rat}}\\ \text{–}\;{\mathrm{Strict}\;\mathrm{Mutation}\;\mathrm{tolerance}}_{\mathrm{Mouse}} \end{array}$$

All the ΔMutTol scores were normalized to obtain ΔMutTol *z*-scores. Proteins with ΔMutTol *z*-scores exceeding 2 times the standard deviation are considered significantly different from each other: (1) proteins with a ΔMutTol z-score > 2 are considered to have higher strict mutation tolerance in naked mole-rat compared with mouse and (2) proteins with a ΔMutTol z-score < −2 are considered to have a lower mutation tolerance in naked mole-rat compared with mouse.

We tested a second definition of mutation tolerance that includes not only neutral mutations (no impact on aggregation propensity), but also mutations that would decrease aggregation propensity (Lenient Mutation tolerance, eq. 8).

(8) $$\mathrm{Lenient}\;\mathrm{Mutation}\;\mathrm{Tolerance}\;\;=\;\frac{\mathrm{Number}\;\mathrm{of}\;(\mathrm{Mutational}\;\mathrm{Agg}\;P\;\leq \;0)}{\mathrm{Total}\;\mathrm{number}\;\mathrm{of}\;\mathrm{mutations}}$$

Additionally, we computed a metric that estimates the proportion of mutations (Proportion of beneficial mutations, eq. 9) resulting in a decrease of aggregation propensity, which we call “beneficial mutations.” For Mutational Agg _P_ scores below −1, we consider a mutation as beneficial.

(9) $$\mathrm{Proportion}\;\mathrm{of}\;\mathrm{beneficial}\;\mathrm{mutations}=\;\frac{\mathrm{Number}\;\mathrm{of}\;(\mathrm{Mutational}\;\mathrm{Agg}\;P\;<\;-1)}{\mathrm{Total}\;\mathrm{number}\;\mathrm{of}\;\mathrm{mutations}}$$

### Pairwise Comparison of Aggregation Propensity and Mutational Aggregation Propensity for ATX Proteins

We first generated the multiple sequence alignment (MSA) of ATX-10 and ATX-3 using Muscle with the default parameters for protein alignment (Edgar 2004). We detected the presence of gaps in the mouse sequence compared to the naked mole-rat sequence. To determine if these gaps correspond to insertion or deletion events, we blasted the naked mole-rat AA sequence across Uniprot database (https://www.uniprot.org/blast/), which were not found with a match in any other rodent species than naked mole-rat (*e*-value < 0.001).

We mapped the per-residue aggregation propensity scores, generated with Tango, to the MSA positions. Similarly, the mutational aggregation propensity scores (Mutational Agg_P_, eq. 5) were also mapped to the MSA positions. Concretely, for a specific AA, we associated the different mutational aggregation propensity score corresponding to all mutated sequence that include a substitution event at this position. If the Mutational Agg_P_ score is above 1, we consider the introduced random mutations to be detrimental, as it increased aggregation propensity. If Mutational Agg_P_ score is below −1, we consider the introduced random mutations as beneficial, as it decreased aggregation propensity.

### Figure Generation and Statistical Analysis

The different plots were generated with Python graphic libraries, Matplotlib (version 3.2.1), Seaborn (version 0.10.0), and Plotnine (version 0.8.0). All statistical analyses were performed using the Scipy stats module (version 1.6.2), unless specified otherwise. The FDR correction was computed with the statsmodels module (0.12.2), unless specified otherwise. Significance thresholds for *P*-values and FDR were set at 0.05. Statistical tests and *P*-values which are reported in the figure legends can be found as outputs of the Python3 scripts that generate the figures.

### Availability of Data and Materials

The processed data and code used to generate the figures are available in the following Github repository: https://github.com/ladyson1806/NKR_lifespan. We also provide the different Python3 scripts and notebooks used to collect and preprocess the initial data set, as well as the code that generates the different scores.

## Supplementary Material

evac057_Supplementary_Data
