# Supplementary material for: Comparative Study of Protein Aggregation Propensity and Mutation Tolerance Between Naked Mole-Rat and Mouse
Source: Genome Biol Evol. 2022 Apr 28;14(5):evac057. doi: 10.1093/gbe/evac057 (PMC9086952; doi:10.1093/gbe/evac057)
Supplement: evac057_Supplementary_Data [file evac057_supplementary_data.zip › 04_Besse_et_al_SM_figures.docx]

# Supplemental Materials

**Comparative study of protein aggregation propensity and mutation tolerance between naked mole-rat and mouse**

Savandara Besse, Raphaël Poujol, Julie G. Hussin

These Supplementary Materials contain the following:

- Supplementary text for Methods
- Supplementary Figure S1 through S3
- Legends for Supplementary Tables S1 through S6

**Supplementary Figure S1**


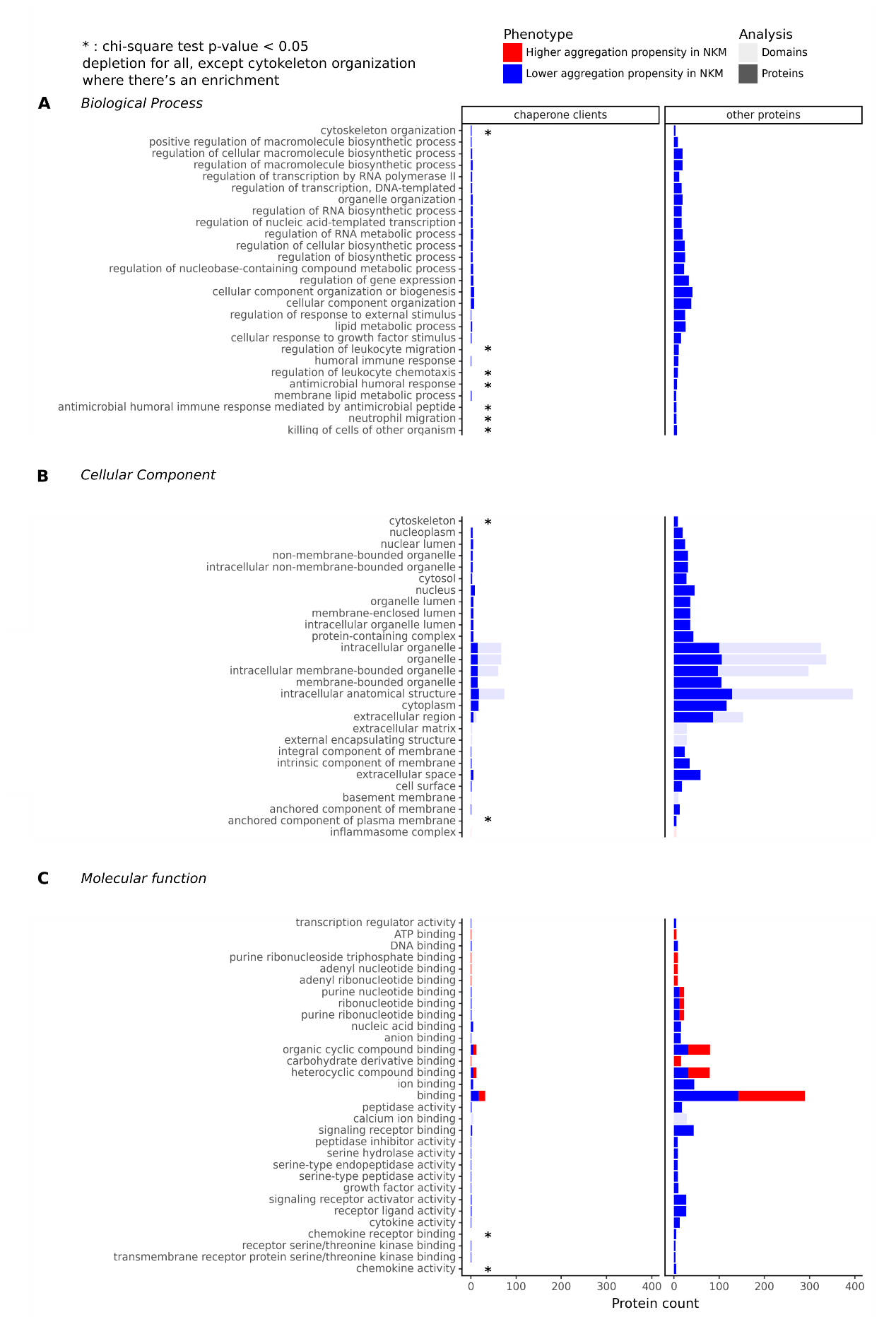


**Supplementary Figure S2**


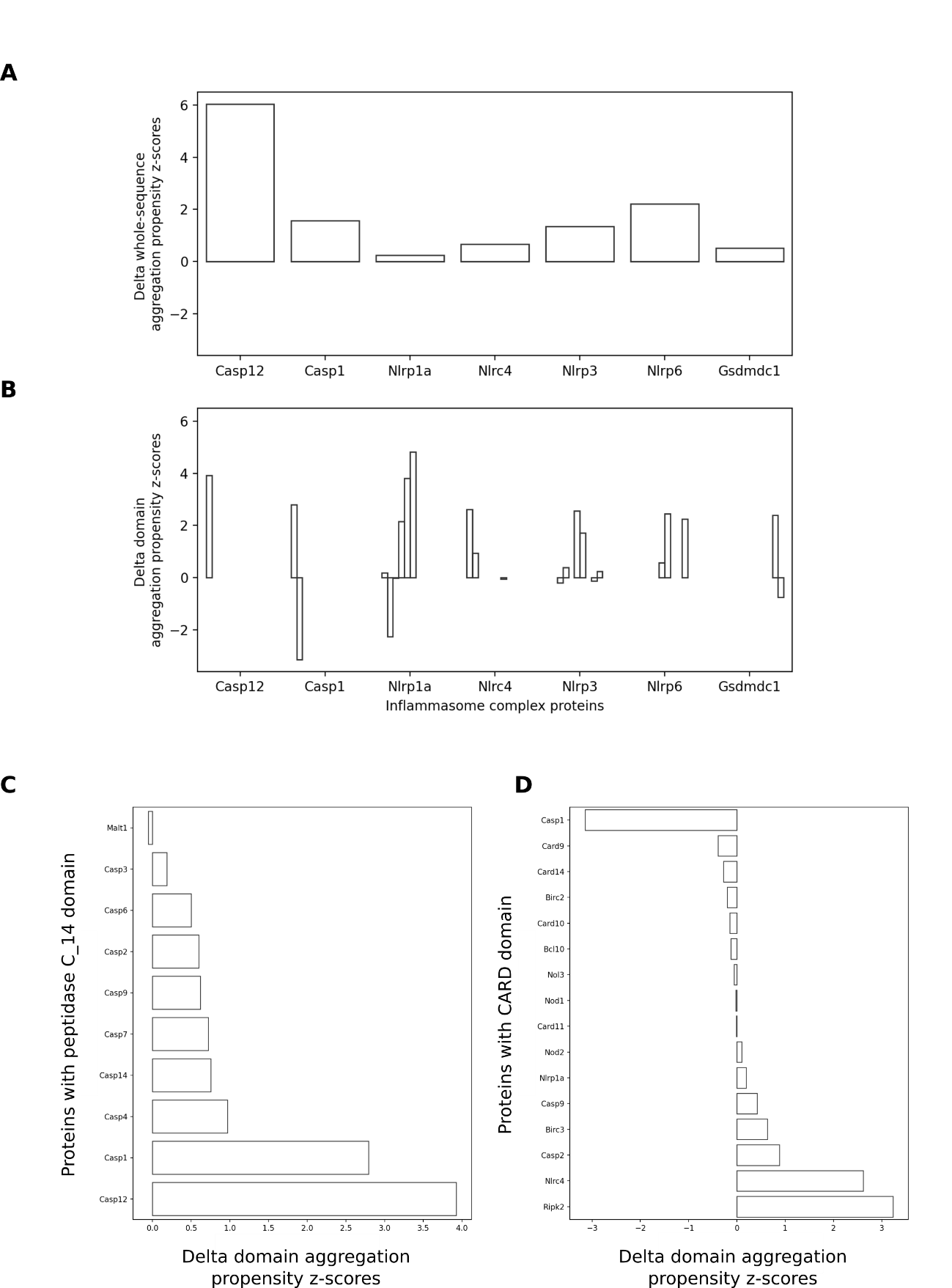


**Supplementary Figure S3
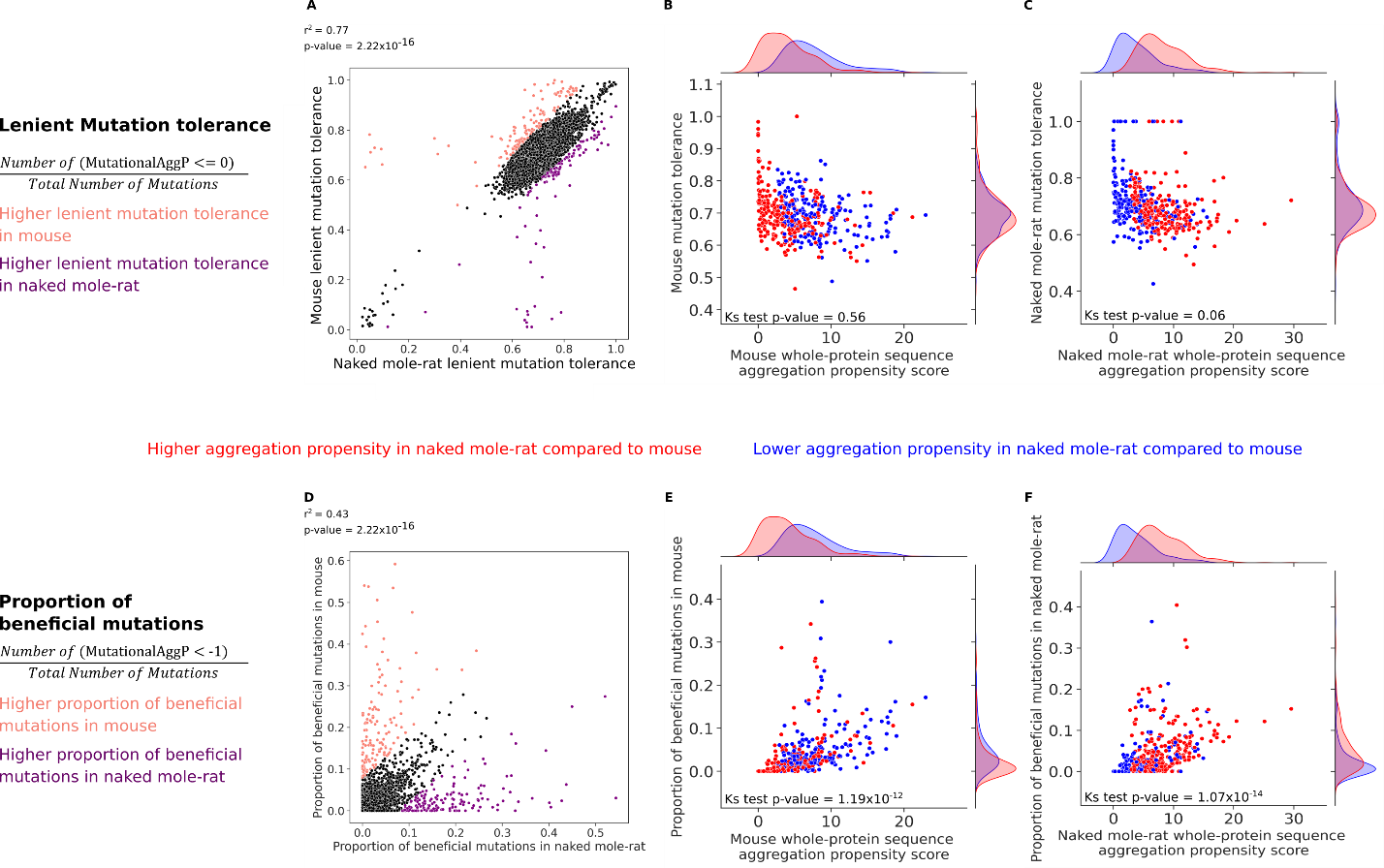
**

**Supplemental Figure legends**

**Supplementary Figure S1: Protein count per GO terms associated to proteins and domains with significant difference of aggregation propensity**

This graph shows the number of proteins within each GO term. Redundant protein identifiers were not removed. (A) shows only chaperone client proteins and (B) shows the rest of the proteins. Subsets of proteins identified with higher aggregation propensity in naked mole-rat compared to mouse are in red and subsets of proteins identified with lower aggregation propensity are in blue. Counts of proteins from the domain analysis have lighter coloration than the counts of proteins from the whole-protein sequence analysis. Only GO terms with at least 5 proteins are shown. The groups of chaperone client proteins with significant differences of distribution compared to the rest of the proteins are marked (*).

**Supplementary Figure S2: Aggregation propensity in inflammasome proteins**

(A) Distribution of the difference of aggregation propensity z-scores between naked mole-rat and mouse in inflammasome proteins at the whole-sequence level. (B) Distribution of the difference of aggregation propensity z-scores between naked mole-rat and mouse in inflammasome proteins at the domain level. The number of bars per protein represents the number of shared domains between naked mole-rat and mouse. (C) Distribution of the difference of aggregation propensity z-scores between naked mole-rat and mouse in proteins with peptidase C14 domain. (D) Distribution of the difference of aggregation propensity z-scores between naked mole-rat and mouse in proteins with CARD domain.

**Supplementary Figure S3: Signatures found from the alternative definition of mutation tolerance and the proportion of beneficial mutations**

(A) Comparison of mutation tolerance (alternative definition) scores in orthologous proteins between naked mole-rat and mouse (n=7,939 proteins). This alternative definition takes account of mutations with no impact in aggregation propensity and the ones that reduce the aggregation propensity. Proteins in the naked mole-rat with higher mutation tolerance are in purple, the ones with lower mutation tolerance are in pink. Scatterplots of mutation tolerance against whole-protein sequence aggregation propensity scores in (B) mouse and in (C) naked mole-rat, restricted to the subsets of proteins identified with significant difference of aggregation propensity (n=510 proteins). Proteins with higher aggregation in naked mole-rat compared to mouse are in red, proteins with lower aggregation are in blue. Kolmogorov–Smirnov (KS) test is used to assess differences in mutation tolerance distributions between the two subsets in each organism. (D) Comparison of proportion of beneficial mutations in orthologous proteins between naked mole-rat and mouse (n=7,939 proteins). Proteins in the naked mole-rat with higher mutation tolerance are in purple, the ones with lower mutation tolerance are in pink. Scatterplots of proportion of beneficial mutations against whole-protein sequence aggregation propensity scores in (E) mouse and in (F) naked mole-rat, restricted to the subsets of proteins identified with significant difference of aggregation propensity (n=510 proteins). Proteins with higher aggregation in naked mole-rat compared to mouse are in red, proteins with lower aggregation are in blue. Kolmogorov–Smirnov (KS) test is used to assess differences in distributions of proportion of beneficial mutation between the two subsets in each organism.

**Supplemental Tables**

All the described supplemental tables are available in the Besse_et_al_SM.xlsx file.

**Supplemental Table legends**

**Table S1: Last updated maximum lifespans and *AnAge* information for Rodents**

This table contains the different metrics used for the generation of Figures 1B,C,D. Details on data collection for Body mass (g), Female maturity (days), Metabolic rate (W), and Data quality are provided in the *AnAge* database (<https://genomics.senescence.info/help.html#anage>). Last updates of maximum lifespans for these species were reported from the list of references available in the last column.

**Table S2: Protein ortholog mapping table between naked mole-rat and mouse**

Mapping table between naked mole-rat (NKR) and mouse (M) orthologous proteins (n=13,806x2). This table was generated from after modification of the *Inparanoid* output where we selected a unique protein pair per ortholog cluster.

**Table S3: GO terms associated with domains with a significant difference in aggregation propensity**

The table was generated based on the result outputs provided by the over-representation analysis performed with the *Panther* database. It is specific to the subsets identified in the domain aggregation propensity analysis, represented by the two sections, higher aggregation propensity, and lower aggregation propensity. Within the sections, each row represents a gene ontology (GO) term associated with the following columns: information on the GO Term (GO Term, GO ID, GO Type), the number of proteins mapped to this GO term in the subset of protein used as background reference (# Reference List), the number of proteins mapped to this GO term in the analyzed subset of protein (# Analyzed List), the number of genes of expected proteins in our subset for this GO term, based on the subset of proteins used as reference (Expected), the ratio of observed number over expected number (Fold Enrichment), their associated raw P-values and FDR. +: Over-representation; -: Under-representation. Rows are sorted by descending values in the Fold Enrichment column.

**Table S4: GO terms associated to proteins with a significant difference of aggregation propensity in naked mole-rat**

The table was generated based on the result outputs provided by the over-representation analysis performed with the *Panther* database. The table is specific to the subsets identified in the per-domain aggregation propensity analysis, represented by the two sections, higher aggregation propensity, and lower aggregation propensity. The descriptions of the columns and rows are like the ones provided for Table S3.

**Table S5: Information of proteins identified in over-representation analysis with significant differences in aggregation propensity**

This table contains the proteins associated with each GO term identified from the functional enrichment analysis. The protein ID column contains the mouse Uniprot identifiers associated with naked mole-rat orthologs and that were used to retrieve their functional annotations, available in the initial FASTA file of the proteins. We also specified to which subsets (chaperone clients or other proteins) these proteins were related.

**Table S6 : Information on inflammasome proteins**

This table provide the information (mouse and naked mole-rat IDs, mouse and naked mole-rat length, gene name and known domain annotations) of the inflammasome proteins of mouse found with a significant difference of aggregation propensity.
